# Robot-Assisted Laparoscopic and Thoracoscopic Surgery: Prospective Series of 186 Pediatric Surgeries

**DOI:** 10.3389/fped.2019.00200

**Published:** 2019-05-21

**Authors:** Mario Navarrete Arellano, Francisco Garibay González

**Affiliations:** ^1^Hospital Central Militar, SEDENA, Mexico City, Mexico; ^2^Department of Pediatrics, Hospital Militar de Especialidades de la Mujer y Neonatología, SEDENA, Mexico City, Mexico

**Keywords:** robotic surgery, pediatric surgery, robotic urological surgery, robotic gastrointestinal surgery, robotic thoracic surgery, robotic oncological surgery, minimally invasive surgery, children

## Abstract

**Objective:** We present the applications and experiences of robot-assisted laparoscopic and thoracoscopic surgery (RALTS) in pediatric surgery.

**Materials and Methods:** A prospective, observational, and longitudinal study was conducted from March 2015 to March 2018 that involved a non-random sample of a pediatric population that was treated with RALTS. The parameters examined were: gender, age, weight, height, diagnoses, surgical technique, elapsed time of console surgery, estimated bleeding, need for hemotransfusion, complications, surgical conversions, postoperative hospital stay, and follow-up. The Clavien-Dindo classification of complications was used. The surgical system used was the da Vinci model, Si version (Intuitive Surgical, Inc., Sunnyvale, CA. U.S.A), with measures of central tendency.

**Results:** In a 36-months period, 186 RALTS cases were performed, in 147 pediatric patients and an adult; 53.23% were male, and the remaining were female. The average age was 83 months, ranging from 3.5 to 204 months, plus one adult patient of 63 years. The stature was an average of 116.6 cm, with a range of 55–185 cm; the average weight was 26.9 kg, with a range of 5–102 kg; the smallest patient at 3.5 months was 55 cm in stature and weighed 5.5 kg. We performed 41 different surgical techniques, grouped in 4 areas: urological 91, gastrointestinal and hepatobiliary (GI-HB) 84, thoracic 6, and oncological 5. The console surgery time was 137.2 min on average, ranging from 10 to 780 min. Surgeon 1 performed 154 operations (82.8%), and the remainder were performed by Surgeon 2, with a conversion rate of 3.76%. The most commonly performed surgeries were: pyeloplasty, fundoplication, diaphragmatic plication, and removal of benign tumors, by area. Hemotransfusion was performed for 4.83%, and complications occurred in 2.68%. The average postoperative stay was 2.58 days, and the average follow-up was 23.5 months. The results of the 4 areas were analyzed in detail.

**Conclusion:** RALTS is safe and effective in children. An enormous variety of surgeries can be safely performed, including complex hepatobiliary, and thoracic surgery in small children. There are few published prospective series describing RALTS in the pediatric population, and most only describe urological surgery. It is important to offer children the advantages and safety of minimal invasion with robotic assistance; however, this procedure has only been slowly accepted and utilized for children. It is possible to implement a robust program of pediatric robotic surgery where multiple procedures are performed.

## Introduction

Robotic surgery is one technology that has gained an enormous surge in use on adults. The general surgical applications have been quite varied in adults, but the technique has been particularly useful in urology for prostate surgery (1–4). There have been few reports that have been published for robotic general pediatric surgery (5–14). Thus, far, the largest number of procedures and publications have been produced for robotic urological pediatric surgery (15–34).

Trends in the literature indicate that pediatric robot-assisted minimally invasive surgery is continuing to be globally utilized (15–23, 31–34). Numerous case reports, case series, and comparative studies have unequivocally demonstrated that robotic surgery in children is safe (35). Robotic enhancements offer improvements to conventional minimal access surgery, permitting technical capabilities beyond existing threshold limits of human performance for surgery within the spatially constrained operative workspaces in children (15).

The first robotic procedure in children was fundoplication, were carried out by Meininger et al. in July 2000 and reported in April 2001 (36, 37).

If traditional laparoscopy is used, the reconstructive procedures are very challenging, and long periods of time are necessary to acquire the appropriate skills and confidence, vs. with RALS (robot-assisted laparoscopic surgery), the learning curve is shorter (31, 38–40).

In systematic investigations of databases of pediatric robot-assisted surgery, the global surgical conversion rate was 4.7% (7), other a net overall reported surgical conversion rate of 2.5% (15). Najmaldin and Antao (41) reported their initial 16-months experience with 50 abdominal procedures involving the gastrointestinal and hepatobiliary (GI-HB) and urological areas. Twelve different robotic surgical techniques were used, and the most frequent surgeries were fundoplication and pyeloplasty, with 6% conversions (41).

In published studies of pediatric robotic surgery, transoperative complications are infrequent, and in the postoperative period, the frequency varies from 0 to 15% (7, 41–43).

Minimally invasive surgery is commonly used for many applications in adult surgical oncology, including biopsy and resection of malignant disease in the chest and abdomen, and management of therapeutic complications. Because there has been an increasing availability of smaller instruments and equipment more suitable to the pediatric patient, for conventional laparo-thoracoscopy, there has been an increase in the use of these techniques with children. With robotic assistance there are also risks, among others, are port-site metastases and peritoneal spread, after resection due to minimal invasion of neoplasms (44, 45). The current status of robotic surgery for tumors in children is low volume usage, and globally a relatively static adoption (46–48).

There is a scarcity of publications from Latin American countries to date describing pediatric patients that have undergone robotic surgery. Secin et al. (49) conducted a survey among the main urologists working in public hospitals in Latin America, in 10 robotic programs based in 4 countries: 4 in Brazil, 3 in Mexico, 2 in Argentina, and 1 in Venezuela. In Venezuela, at the University Hospital of Caracas, 4 robotic surgeries had been performed in pediatric patients, with 2 pyeloplasties, one megacolon surgery, and one resection of an ovarian tumor that had been treated in the period from 2010 to 2012 (unpublished data). For adult patients, there are publications for various specialties (50–54).

The objective of this publication is to present the applications and our experience of RALTS in pediatric surgery.

## Materials and Methods

This prospective, observational and longitudinal study of the robotic surgeries performed on a pediatric population was conducted from March 2015 to March 2018. Our hospital is a public tertiary care facility, and the robotic surgery program includes several surgical specialties in adult and pediatric surgery. The diagnoses of the pathologies were made with laboratory, radiological, cabinet, and special studies according to the case.

Non-random samples were all treated with RALTS. The parameters recorded were gender, age, weight, height, diagnoses, surgical technique, elapsed time of console surgery, estimated bleeding, hemotransfusion, complications, conversions, postoperative hospital stay, and follow-up. Both authors (surgeons) initially performed some simple surgical techniques, and then advanced to more complex surgeries, with subsequent advancement to different points of the learning curve for robotic surgery.

The Clavien-Dindo classification of surgical complications was used (55, 56). The surgical system used was the da Vinci model, Si version (Intuitive Surgical, Inc., Sunnyvale, CA. U.S.A). We used 8-mm robotic instruments consisting of 2 or 3 robotic trocars according to the surgical technique, 8.5-mm or 12-mm robotic 30° lens for a three-dimensional camera, and a 5-mm trocar laparoscopic for one assistant.

In the following procedures, we used 3 robotic work arms: for urological surgery, in the Mitrofanoff procedure with or without augmentation cystoplasty; for GI-HB surgery, in fundoplications and in the biliodigestives; and in thoracic surgery, in lobectomies, and in oncological surgery, in 4 of the 5 procedures, the exception was the resection of the mediastinal teratoma. In the first 10 fundoplications, we used a 1-0 silk suture to elevate the liver, but after lesion of the left hepatic duct occurred, and we subsequently opted to use the third robotic instrument for that purpose. The docking charts for robotic surgery that are suggested for surgical techniques in adults were not applicable for children. Thus, at times, 3 cm of separation was required between each trocar when performing surgery on infants, due to the limited space in such a small patient.

The postoperative follow-up was at 8, 30, and 90 to 120 days, and then every 6 months. Between 90 and 120 days, radiographic and cabinet studies were carried out according to the treated pathology to evaluate the results. We used measures of central tendency. The analysis of the results was made based on the number of procedures. The data was entered into a spreadsheet in Microsoft Office Excel 2013 version.

In relation to ethical considerations of the study, being of an observational nature, it was not necessary to consent to enter the study to the patients. The Research Ethics Committee of the Hospital evaluated and approved the study. In Mexico, robot-assisted surgery complies with the records and regulations of the Mexican health authorities. In our institution, robotic surgery is routinely authorized for execution. In order to perform the medical-surgical procedures, we obtained the informed consent in writing from the parents or guardians and the adult patient.

## Results

In a 36-months period, we performed 186 RALTS in 147 pediatric patients and one adult patient. Of the procedures, 53.23% (99) were in male, and the rest were female; the average age was 83 months, ranging from 3.5 to 204 months, plus one patient of 63 years. The average height was 116.6 cm, and ranged from 55 to 185 cm, with an average weight of 26.9 kg, ranging from 5 to 102 kg; the smallest patient was 3.5 months old with a height of 55 cm and weight 5.5 kg (Supplementary Table 1).

We performed 41 different surgical techniques, grouped in 4 areas: urological 91 (48.92%), GI-HB 84 (45.16%), thoracic 6 (3.23%), and oncological 5 (2.69%), as shown in Table 1. Our 3 most frequent urological procedures were pyeloplasty, nephrectomy, and ureteral reimplantation, totaling 54 and representing 59.34% of this area. GI-HB, primary fundoplication, redo-fundoplication, and cholecystectomy totaled 62 and represented 73.8% in this area. Diaphragmatic plication and lobectomy were the most frequent for thoracic surgery, and there were only isolated cases of oncological surgery.

**Table 1:** Prospective series of 186 pediatric surgeries using RALTS.

**Area**	**Procedures**	***n* (%)**
**UROLOGICAL**		
	Pyeloplasty	19
	Nephrectomy	18
	Ureteral reimplantation	17
	Mitrofanoff	6
	Nephroureterectomy	6
	Varicocelectomy	5
	Release of extrinsic obstruction	
	UP union	3
	Inguinal hernia repair	3
	Desderivation of ureterostomy and ureteral reimplantation	2
	Cystolithotomy	2
	Various^*^	10
	**Subtotal**	**91 (48.92)**
**GI-HB**		
	Primary fundoplication	38
	Redo fundoplication	13
	Cholecystectomy	11
	Gastrostomy	9
	Biliodigestive^**^	6
	Operation of malone	2
	Various^***^	5
	**Subtotal**	**84 (45.16)**
**THORACIC**		
	Diaphragmatic plication	3
	Lobectomy	2
	Bronchogenic cyst excision	1
	**Subtotal**	**6 (3.23)**
**Oncological**		
	Mediastinal teratoma	1
	Resection of carcinoid in stomach	1 (adult)
	Left radical nephrectomy	1
	Retroperitoneal lipoma	1
	Left adrenalectomy (pheochromocytoma)	1
	**Subtotal**	**5 (2.69)**

* *Ureteroureterostomy, augmentation cystoplasty, bladder neck closure, heminephrectomy with ureterectomy, perirenal abscess drainage, colostomy closure, enterovesical fistula closure, review of Mitrofanoff, ureterostomy, and ureteropyelography*.

** *Roux-en-Y hepaticojejuno (5) or portojejuno (1) anastomosis reconstruction*.

*** *Duodenoplasty with adherensiolysis, extraction of gastric trichobezoar, drainage, and debridement of recurrent retrohepatic abscess post-appendectomy, gastrojejunoanastomosis with Roux-en-Y and splenectomy*.

Of the total procedures, the elapsed console surgery time was an average of 137.2 min, ranging from 10 to 780 min. Surgeon 1 performed 154 procedures (82.8%), and Surgeon 2 performed 32 procedures (17.2%). Global hemotransfusion occurred in 4.83%, and complications occurred in 2.68%, with a surgical conversion rate of 3.76%. The average postoperative stay was 2.58 days, and the average follow-up was 23.5 months.

There was a predominance of patients males in urological (62.6%), and oncological (80%), and a predominance of patients females in thoracic (66.6%) and GI-HB (53.5%). In relation to age, weight, and height, the smallest group of patients underwent thoracic procedures (14.5 months, 9.1 kg, 78.8 cm, average values), and the largest underwent GI-HB surgery (97.7 months, 29.7 kg, 125 cm, average values), intermediate average values those subjected to urological surgery (72.9 months, 20.3 kg, 110 cm), and oncological patients (57.3 months, 18 kg, 113 cm, average values). Patients of 10 kg or less were 12 of urological, 10 of GI-HB, 4 of thoracic and 1 of oncological procedures, totaling 27 cases, representing 14.5% of our casuistry.

The evolution of the console surgery times is exemplified by the most frequently performed procedures, which were urological, pyeloplasty, and GI-HB, primary fundoplication. Surgeon 1 consistently reduced the time required for surgery as the procedures continued to evolve and advance, of 227 min in the first pyeloplasty at 115 min in the procedure 19, and for primary fundoplication, it was reduced of 115 min in the first fundoplication at 56 min in the procedure 26 (Supplementary Figures 1, 2).

Of the three most frequently performed procedures by area, the following results were obtained. For urological procedures, 19 pyeloplasty surgeries were performed, with console surgery time of 183 min on average, 0% conversions, 5.2% complications (1 case), stay PO of 3.4 days, with a success rate of 100%. There were 18 nephrectomy surgeries, with console surgery time of 102 min on average, 0% conversions, 5.2% complications (1 case), and stay PO of 1.9 days. There were 17 units of ureteral reimplantation, with console surgery time of 139 min on average, 0% conversions, 0% complications, stay PO of 2.1 days, with 88.24% resolution of reflux; one patient with recurrent bilateral reflux was reoperated and reflux was resolved, with 100% secondary success.

Appendicovesicostomy or Mitrofanoff operation, we performed 6 of these procedures, without predominance of gender, with average age of 9.25 years, weight of 30.7 kg, height of 1.24 m, console surgery time of 262 min, estimated bleeding of 26 ml, with one conversion, and stay PO of 4.34 days. For one patient at 30 days PO, it was improperly manipulated and the Mitrofanoff was dismantled. This complication was not related to surgery, and thus, we can consider a success of 83.34%; open surgery was used for the reoperation, and at the follow-up, all the ducts were continent.

For GI-HB surgery, there were 38 primary fundoplication procedures, plus gastrostomy for feeding in 9 cases, with a console surgery time of 159 min on average, 5.26% conversions (2 cases), 5.26% complications (2 cases), stay PO of 2.4 days; at follow-up PO, there were 2 cases of partial dismantling of fundoplication and hiatal hernia (5.26%), at 11 and 24 months, with a follow-up PO of 17.7 months on average. There were 13 procedures of redo fundoplication (the two robotic cases with recurrence are included), with a console surgery time of 188 min on average, 7.7% conversions (1 case), 0% complications, and stay PO of 2.3 days, with a follow-up PO of 19.5 months on average. There were 11 cholecystectomy procedures, with a console surgery time of 53 min on average, 0% conversions, and 0% complications, with a stay PO of 1.5 days.

The results of 4 patients treated with choledochal cyst, 3 females and 1 male, with average age of 36 months, average weight of 15 kg, and average height of 94.2 cm. There were three cases of cyst type 1-A and one type 1-C; the size of the cyst was 8–12.5 cm, with an average of 9.6 cm. Minimally invasive surgery was performed, with the extracorporeal Roux-en-Y procedure assisted by laparoscopy, and resection of the cyst, cholecystectomy, and the hepaticojejunal anastomosis to Roux-en-Y was performed with robotic assistance. The average surgical times were 130 min with assistance by laparoscopy and 230 min for console surgery time, with an average of 32.5 ml of bleeding. There were no conversions or complications. The average PO stay was 4.7 days, with a 15-months follow-up, and the patients evolved asymptomatically.

For thoracic procedures, there were 3 procedures with diaphragmatic plication, with a console surgery time of 161.6 min on average, 0% conversions, 33.3% complications (1 case), and stay PO of 5.6 days. There were 2 lobectomy procedures, with a console surgery time of 314 min, 50% conversions (1 case), 0% complications, and stay PO of 3 days. There was 1 procedure of resection of a bronchogenic cyst, with console surgery time of 269 min, with no conversion, no complications, and stay PO of 3 days.

There were 5 isolated cases of oncological procedures (Table 1).

The estimated average bleeding in each area of procedures performed, in relation to the average weight of the patients, was as follows: blood loss was 6.1 ml/kg for oncological procedures, 2 ml/kg for thoracic, 1.3 ml/kg for urological, and 1.1 ml/kg for GI-HB. However, the highest number of hemotransfusions occurred during the GI-HB procedures, with 5 cases, against 4 cases of the remaining 3 areas of procedures.

There were 2 complications that occurred intraoperatively (IO) a lesion of the left hepatic duct when applying a suture point for traction and elevating the liver during a fundoplication, and rupture of a renal vein when trying to apply a staple during a nephrectomy. These complications were classified as IIIB and II, respectively, according to the Clavien-Dindo classification. The treatment of these complications was Roux-en-Y hepatico-yeyunostomy, and bleeding control and hemotransfusion, respectively.

There were 3 PO complications: (i) a urinoma in a patient with pyeloplasty, with readmission at 9 PO days, (ii) an enterovesical fistula in 2a., 1 week PO, in a patient with augmentation cystoplasty and colostomy closure, and (iii) prolonged drainage of pleural fluid (11 days) in a patient with diaphragmatic plication. According to the Clavien-Dindo classification, the first two corresponded to IIIb, and the third corresponded to I. The treatment of these complications consisted of percutaneous application of a multiproposite catheter for the first patient, a second surgery was performed for the second patient, and the last patient was hospitalized for 12 days to drain fluids.

The robotic procedures that required conversion, was to an open surgery. In the urological procedure, the conversion was required due to the technical difficulty in the appendicovesicostomy anastomosis. In the GI-HB procedures, the reasons for the 4 conversions were multiple intestinal adhesions, technical difficulty in 2 primary fundoplications with gastrostomy, and in a redo of a fundoplication. The reason for the conversion into a lobectomy was technical difficulty, and in the radical nephrectomy, it was difficult to identify the anatomy when dividing the horseshoe kidney.

The longest average PO stay of 4.33 days occurred in patients that underwent a thoracic procedure. In the other 3 areas, the PO stays were 2.48 days in GI-HB, 2.58 days in urological and 2.6 days in oncological. The average PO follow-up among the patients in the 4 areas of surgical procedures was very similar, between 24.5 and 31.4 months, with a range between 7 and 43 months overall (Supplementary Table 2).

## Discussion

The robotic surgery program began in our hospital in November 2014, and pediatric surgery was incorporated in March 2015. The first procedures with robotic assistance were performed on the pediatric population on March 23, 2015, after Surgeon 1 received training and certification as a console surgeon of the da Vinci surgical system, with previous experience in open surgery and conventional laparo-thoracoscopy. Recommendations were followed to perform some less complex cases and progress toward highly complex procedures, with subsequent integration into the Hospital Committee of Robotic Surgery.

In our experience, the order of frequency of the procedures, from highest to lowest by area, was urological, GI-HB, and thoracic (Table 1), which coincides with what has previously been reported (15). There are numerous reports that the most frequent urological procedure performed is pyeloplasty, and the most frequent GI-HB surgery performed is fundoplication, which varies between lobectomy, ligation of the ductus arteriosus, and mediastinal masses in the reports of thoracic procedures, which are aspects that also coincide with our treated pediatric population (5–7, 15).

During the 18 years that have elapsed since the first 2 fundoplications were carried out (36, 37), more than 70 different surgical techniques have been published. Cundy et al. performed a 2013 systematic literature search for all reported cases of robotic surgery in children during an 11-year period. During this time, 137 articles reported 2,393 procedures in 1,840 patients, and the most prevalent gastrointestinal, genitourinary, and thoracic procedures were fundoplication, pyeloplasty, and lobectomy, respectively (15). These 3 previous robotic surgical techniques represented 46.55% and genitourinary procedures 59.92% of the total surgeries, confirming that the greatest amount of these types of published studies are in the field of urology (16–34).

In 11-years period (April 2001 to March 2012), the published literature reporting robotic surgery in children, categorized by study design, comprises 34% case reports, 52% case series, and 14% non-randomized comparative studies (*n* = 220 publications), and the study design was prospective in only 6% of non-randomized comparative studies. As well, 79% of these 220 publications originated from the United States, and the remainder were from 17 other countries, with 14% from Europe, 4% from the Middle East, and 3% from Asia (15).

Qualitatively, we find that with complex and laborious procedures, the time of console surgery is longer as compared to standard open surgery, but we agree that robotic surgery enables more refined hand-eye coordination, superior suturing skills, better dexterity, and precise dissection. It is achieved by the characteristics of robotic surgical platforms that include motion scaling, greater optical magnification, 3D and stereoscopic vision, increased articulated instrument tip dexterity, tremor filtration, operator-controlled camera movement, and elimination of the fulcrum effect (15), and all of this translates into greater safety for patients and advantages for the surgeon.

Considering the examples of 19 procedures of pyeloplasty (with an average of 183 min) and 38 procedures of primary fundoplication plus gastrostomy for feeding in 9 cases (an average of 159 min) as the most frequently performed procedures, our console surgery times are very satisfactory when comparing them with what was reported by other authors as 221 and 170 min, respectively, without including added procedures (7). In a meta-analysis of fundoplication, 6 series of patients were included for a total of 135 patients that underwent robotic fundoplication surgery (with some cases of gastrostomy), and the average console surgery time was 168.3 min, with 3% conversions, 8.9% complications, and 5.31 days PO stay (57).

Robotic ureteral reimplantation for the treatment of pediatric vesicoureteral reflux should be reclassified as a complex reconstructive procedure in pediatric urology. Over the past decade, higher than expected complication rates and suboptimal reflux resolution rates at some centers have been reported. The robotic ureteral reimplantation results have widely varied, with reflux resolution from 77 to 100% and complications from 0 to 12.5% (58). Using a standard technique to improve reimplantation results that was modified according to Gundeti et al. (59), we obtained results with 0% complications, primary success of 88.24%, and secondary success of 100%.

Although it is recommended that a surgeon perform a minimum of one robotic operation per week, it is important to emphasize that 15 to 30 robotic procedures should be performed to achieve optimal console surgery times, according to the type of procedure. Then, the use of this technology can become profitable. The period during which a surgeon finds that the procedures are more difficult, take longer, and there is potentially a higher rate of complications and less effectiveness due to inexperience, which is called the learning curve (60). The use of robot assistance dramatically decreases the learning curve, because overcome the limitations of conventional laparoscopy (31, 38–40). Our console surgery times have evolved in 19 pyeloplasties and in 26 fundoplications, with a decrease of 50% and of almost 52%, respectively.

Robotic assistance has special applications in complex and reconstructive surgery. By areas, urological: pyeloplasty, ureteral reimplantation, augmentation cystoplasty, and Mitrofanoff procedure; GI-HB: revision fundoplication and hiatal hernia and biliary-digestive correction; thoracic tumors: tumor excision and thymectomy; and oncological: resection of tumors of selected cases. For all these procedures, from the open technique, we jump to robotic surgery.

Because of the limitations, conventional laparoscopic surgery in pediatric surgery has been primarily limited to simple or extirpative surgery, more complex or reconstructive surgery by laparoscopy, can only be performed by a limited number of highly qualified surgeons (61).

Since the publication of the initial experience of robotic-assisted laparoscopic ileocistoplasty and the appendicovesicostomy of Mitrofanoff by Gundeti et al (62), it was shown that is a safe, feasible and effective procedure (62). Of the urological surgery, is one of the most complex procedures, in a publication of the retrospective cohort consisted of 18 patients, with mean age of 11 years, console surgery time of 494 min, stay PO of 5.2 days, continence in 94.4%, Clavien-Dindo grade 1 complications in 5 patients and grade IIIb in 2 patients (63).

The patients with neural tube defects may also require redo surgery at the bladder neck for persistent incontinence or any of these procedures with creation of a Malone antegrade continence enema (64, 65).

In a series of cases, retrospectively evaluated for open appendicovesicostomy and robotic, the comparison did not reveal significant differences in the number of acute complications or reoperations between the groups (66).

Our results are satisfactory with the Mitrofanoff, of 6 procedures, one conversion, and all the ducts were continent to follow up.

A multi-center analysis was performed in the United States regarding the complications and conversions in a large cohort of pediatric patients (880 procedures) that underwent robotic urological surgery to 90-day PO. There were with 41 (4.8%) Grade IIIa and Grade IIIb complications, and one patient (0.1%) had a grade IVa complication. Intraoperative visceral injuries secondary to robotic instrument exchange and traction injury were seen in four patients (0.5%), with subsequent conversion to an open procedure. Grade I and II complications were seen in 59 (6.9%) and 70 (8.2%) patients, respectively. The overall 90-day complication rate was similar to those appearing in reports of laparoscopic and open surgical procedures. A total of 14 (1.6%) surgeries were converted to an open or pure laparoscopic procedure (67).

Complications of robotic surgery in urology in a single institution, with 10 different types of procedures performed in included 136 patients, 11 total complications (8.1%): 2 grade I (1.5%), 7 grade II (5.1%), and 2 grade IIIb (1.5%). Complications included ileus in 2 patients, port site infection in 2, urinary leak in 2, urinary retention in 2, urinary tract infection in 2, and stent migration in 1 (43).

There are few reports of minimally assisted surgery by robot for the treatment of choledochal cyst (68–72). Currently, the standard treatment is open surgery. The first cases of resection of choledochal cyst with robotic assistance were reported by Lee et al. (70) and Woo et al. (71).This treatment method is safe and effective, it is associated with earlier postoperative feeding and discharge from the hospital, technically robotic-assistance facilitates performing the biliodigestive anastomosis and for pediatric choledochal cyst showed results comparable to those for open surgery, and thus, is considered to be a valid and alternative surgery for this pathology (71, 72).

A recent systematic review that included a total of 86 patients, 7 patients experienced conversion to open surgery, and the surgery success rate was 91.9%. The hospitalization time was 8.8 days. Eight patients had biliary fistula, one patient had anastomotic stenosis, and one patient had wound dehiscence (73).

The results in the 4 cases that we treat of choledochal cyst confirm the safety and efficacy of this surgical alternative, with 0% complications and conversions.

There are benefits and limitations of using robotic surgery for children with cancer (74), it is feasible for the surgical treatment of tumors in pediatric patients and only isolated adverse events have been reported for malignant tumors, such as tumor spillage and residual disease (45). The current status of robotic surgery for tumors in children is low volume usage, in a relatively static global state of adoption and when applied the oncological surgical principles must be respected (46, 47).

In pediatrics, urologic oncology cases are often managed with open surgery, but is feasibility of using the robotic approach in carefully selected cases, and safely and effectively adapted adult robotic techniques for genitourinary oncology cases in children and young adults (48).

Our experience with robotic-assisted surgery for oncological procedures is limited, with only isolated cases to date. However, the results have been satisfactory, with only one conversion and 0% complications in 5 cases. It is only a matter of time before appropriate cases arise, and we will be going forward in this area with robotic surgery.

Hemotransfusion occurred in 4.83% of the procedures we performed. The average calculation per/kg of weight of estimated bleeding was minimal, and the highest number of hemotransfusions was in the GI-HB procedures, with 5 cases consisting of 4 fundoplications and the case of duodenoplasty with adherensiolysis that merited conversion to open. Complications occurred in 2.68% (5 cases), two IO and three PO. In published reports, rates are very variable, with complications occurring in 8 cases (3%) of 274 robotic procedures during the first 6 years of experience in a London hospital (42). Other reported with 50 abdominal procedures involving the gastrointestinal and hepatobiliary (GI-HB) and urological areas, twelve different robotic surgical techniques were used, there was 6% conversions (41).

In systematic investigations of databases containing information on robot-assisted surgery in children, the global complications ranged from 0 to 15% (7).

Our global conversion rate of 3.76% did not significantly differ from that reported by Cundy et al. (15), with 2.5% overall; by area of procedures, the conversion rates of gastrointestinal, genitourinary, and thoracic procedures were 3.9, 1.3, and 10%, respectively (15). Our conversion rates of the same areas were 4.76, 1.09, and 16.6%, respectively. In their first 100 robotic surgeries, Meehan and Sandler (5) reported conversions of 13.48% in non-urological abdominal procedures and 18.18% in thoracic surgeries. For 96 robotic procedures, De Lambert (2013) reported a conversion rate of 3.1%, which corresponds to patients in the groups of general surgery, urologic surgery, and thoracic surgery (6). A systematic database search was performed that included data from all published reports until October 2007 (31 studies and 513 patients), and the conversion rate for all pediatric robotic procedures was 4.7% (7).

We have not been presented with malfunctions of the robot during the procedures.

There are very few publications of pancreatic pathology in children, treated with a robotic approach, we find only case reports (75–77).

There are approximately 60 da Vinci systems in hospitals in Latin America to date, and there are few that are used to perform surgery on children. The costs of technology, consumables, and maintenance are the main obstacles in Latin American countries that prevent robotic surgery from being practiced more widely. The situation is more adverse in private hospital institutions, because insurance companies only approve a few cases of robotic surgery for adult patients, and children are regularly not approved.

We consider viable, if there is enough experience in Pediatric Surgeons, to implement a robust program of robotic surgery where multiple procedures are performed and after overcoming the learning curve, with efficiency, effectiveness and safety to be used in the complex cases of the different areas of Pediatric surgery is a way to maintain the volume of cases and reduce costs by using robotic assistance routinely in conjunction with other surgical specialties.

The biggest obstacles preventing the use of robotic surgery on a pediatric population are the learning curve, technical limitations, the size of the robotic instruments, and the inconvenience, the costs (78, 79). The manufacturer of the da Vinci surgical robot recommends an 8-cm distance between each port. This is impossible to achieve in neonatal cases (79). This technical limitation, we overcome it have used a 3-cm separation between each trocar, and we have performed various procedures in younger infants with no problems.

There are very few studies that evaluate its cost (30) in an integral way and consider a multitude of factors in addition to the surgical event. The most important factor in Latin American countries is cost, which limits the adoption of robotic surgery.

The outcomes of RALTS are comparable to open surgery and conventional laparoscopic surgery and demonstrate continued improvement with experience. Outcomes can become more cost-efficient if shorter operative times are achieved, and with marketing competition, by providing less expensive robotic systems and instruments. Robotic surgery is suitable in the pediatric practice, which necessitates fine dissections and sutures in narrow anatomical spaces. The results of robotic surgery in the field of pediatrics are encouraging (80).

Our data and other series demonstrate that the short- and long-term morbidity associated with robotic surgery is low in a pediatric population, even during the learning period (44). This information allows affirmation that robot-assisted laparoscopic surgery is safe and efficient in the pediatric population. Although open surgery is still the gold standard for many pediatric diseases, there is a chance to change this view due to the inherent advantages in reconstructive surgery that can be attained with robot-assisted laparoscopy (81). We fully share this vision and consider that children, like adults, should have the opportunity and receive the benefits of RALTS.

## Conclusion

RALTS is safe and effective in children. An enormous variety of surgeries can be safely performed including complex surgical cases, even in small children. There are few published prospective series describing pediatric RALTS, since most are only urological.

This technology has only been slowly adopted for use in children. Pediatric Surgeons must advocate for the benefit of our patients and overcome the obstacles to increase the adoption and more widely disseminate its use.

The present prospective series reporting pediatric robotic surgery is the first in Latin America, and its results are very satisfactory, which allows us to affirm that we have accumulated favorable experiences and that we can offer our pediatric patients the benefits and advantages of robotic surgery.

It is possible to implement a robust program of pediatric robotic surgery where multiple procedures are performed.

## Author Contributions

MNA contributed to the prospective registration of all the information, designed the data spreadsheet for Microsoft Office Excel 2013 version, performed 154 procedures, performed patient follow-up, analyzed the published information (references), analyzed the experimental results, and compared them with the published data, and wrote the manuscript. FGG contributed information to the prospective registry pertaining to the 32 procedures that he performed, and also obtained follow-up information from the patients.

### Conflict of Interest Statement

MNA declares to be Proctor of the da Vinci Surgical System and sometimes receives salary for advice to Surgeons in their first robotic procedures, from the marketing company in my country, as part of the support in the training of Surgeons by this company. But, in relation to the treatment of patients and the execution of this manuscript, no economic financing was received from commercial companies. The remaining author declares that the research was conducted in the absence of any commercial or financial relationships that could be construed as a potential conflict of interest.
