# Supplementary material for: Robot-Assisted Laparoscopic and Thoracoscopic Surgery: Prospective Series of 186 Pediatric Surgeries
Source: Front Pediatr. 2019 May 21;7:200. doi: 10.3389/fped.2019.00200 (PMC6537604; doi:10.3389/fped.2019.00200)
Supplement: Supplementary file 1 [file Table_1.pdf]

Supplementary table 1. Demographic data of the patients by area of RALTS patients in pediatric surgery "Prospective series of 186 surgeries"

Area	Procedures (%)	Gender (%) Male	Gender (%) Female	Age* Months	Weight* Kg	Height* cm
Urological	91 (48.92)<br>63 Patients**	57 (62.64)	34 (37.36)	72.9<br>4-204	20.32<br>6.2-75	110<br>55-172
GI-HB	84 (45.16)<br>74 Patients**	39 (46.43)	45 (52.57)	97.76<br>3.5-204	29.75<br>5-102	125<br>57-185
Thoracic	6 (3.23)<br>6 Patients**	2 (33.33)	4 (66.67)	14.5<br>8-24	9.1<br>6-12	78.8<br>66-88
Oncological	5 (2.69)<br>5 Patients**	4 (80)	1 (20)	57.33<br>28-72	18<br>9.5-22.5	113<br>87-128

*Age, weight and height, average value and range.
**Total patients 148, in 38 patients, 2 procedures were performed.
