# Supplementary material for: Robot-Assisted Laparoscopic and Thoracoscopic Surgery: Prospective Series of 186 Pediatric Surgeries
Source: Front Pediatr. 2019 May 21;7:200. doi: 10.3389/fped.2019.00200 (PMC6537604; doi:10.3389/fped.2019.00200)
Supplement: Supplementary file 2 [file Table_2.pdf]

Supplementary table 2. Results of the procedure variables by area of RALTS in pediatric surgery "Prospective series of 186 surgeries"

	Console surgery time*	Estimated bleeding*	Hemotransfusion**	Complications**		Conversion**	stay PO*	Follow-up*
	Range	Range	n	IO***	PO***	n	Range	Range
**Urological (n 91)**								
	159 minutes	26.4 ml	2.19 %	1.09 %	2.19 %	1.09 %	2.58 days	27.93 months
	10-408	0-200	2	1	2	1	1-11	9-43
**GI-HB (n 84)**								
	145 minutes	33.4 ml	5.95 %	1.19 %	0 %	4.76 %	2.48 days	24.5 months
	23-435	0-350	5	1		4	1-13	7-43
**Thoracic (n 6)**								
	182.6 minutes	18.3 ml	16.6 %	0 %	16.6 %	16.6 %	4.33 days	24.5 months
	28-314	5-40	1		1	1	2-12	11-39
**Oncological (n 5)**								
	132.2 minutes	110 ml	20 %	0 %	0 %	20 %	2.6 days	31.4 months
	90-194	0-250	1			1	2-4	15-39

* Average value and range. ** Percentage or index and n of cases. *** IO and PO, intraoperative and postoperative.
