# Supplementary figures and images for: Robot-Assisted Laparoscopic and Thoracoscopic Surgery: Prospective Series of 186 Pediatric Surgeries
Source: Front Pediatr. 2019 May 21;7:200. doi: 10.3389/fped.2019.00200 (PMC6537604; doi:10.3389/fped.2019.00200)

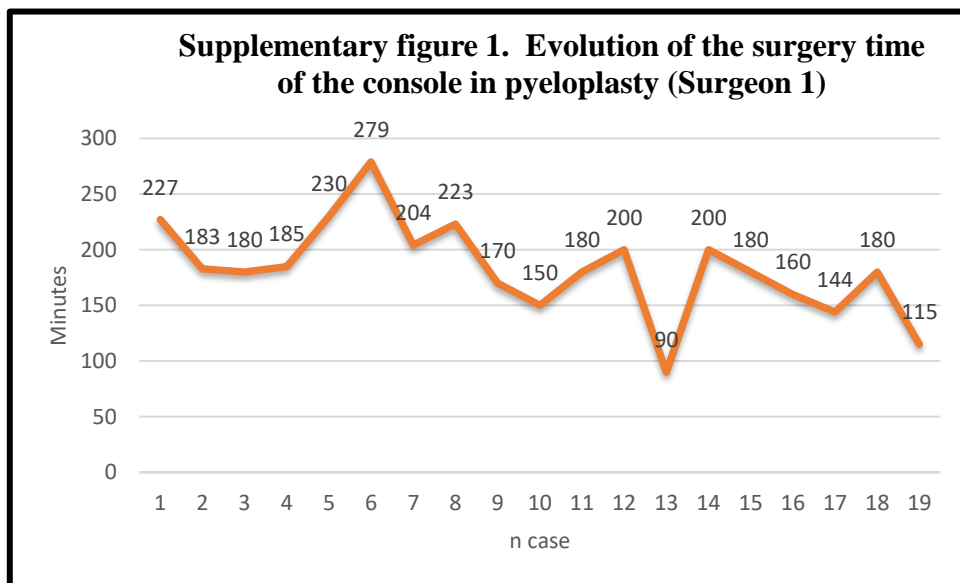

Note: Average console surgery time 183 minutes.

Supplement: Supplementary file 3 [file Data_Sheet_1.pdf]

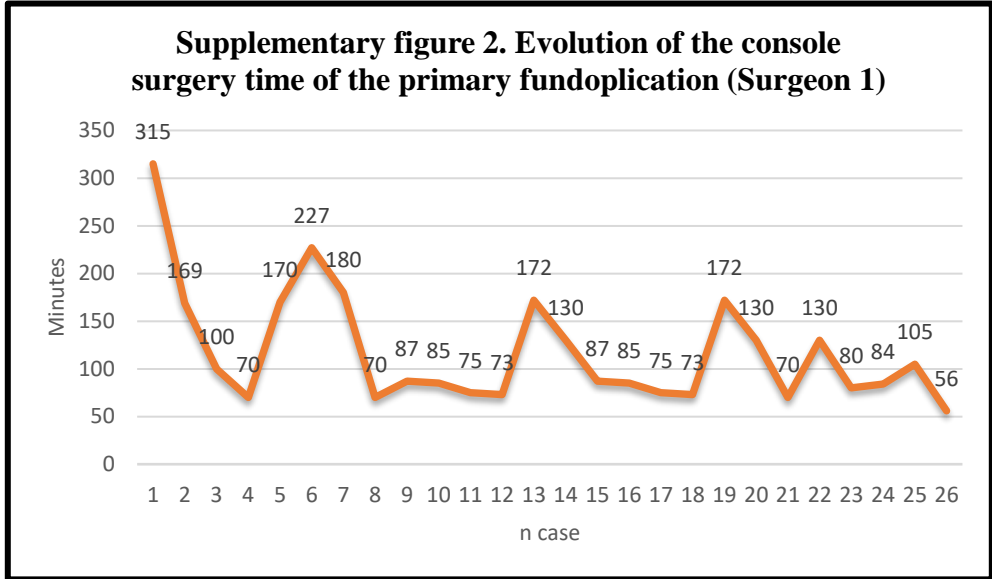

Note: Average console surgery time 118 minutes.

Supplement: Supplementary file 4 [file Data_Sheet_2.pdf]
